# Misinformation About COVID-19 in Sub-Saharan Africa: Evidence from a Cross-Sectional Survey

**DOI:** 10.1089/hs.2020.0202

**Published:** 2021-02-18

**Authors:** Uchechukwu L. Osuagwu, Chundung A. Miner, Dipesh Bhattarai, Khathutshelo Percy Mashige, Richard Oloruntoba, Emmanuel Kwasi Abu, Bernadine Ekpenyong, Timothy G. Chikasirimobi, Piwuna Christopher Goson, Godwin O. Ovenseri-Ogbomo, Raymond Langsi, Deborah Donald Charwe, Tanko Ishaya, Obinna Nwaeze, Kingsley Emwinyore Agho

**Affiliations:** Uchechukwu L. Osuagwu, PhD, is a Research Fellow, Diabetes, Obesity and Metabolism Translational Research Unit, School of Medicine; and Kingsley Emwinyore Agho, PhD, is an Associate Professor of Biostatistics, School of Health Sciences; both at Western Sydney University, Campbelltown, Australia. Chundung A. Miner is an Associate Professor, Department of Community Medicine, College of Health Sciences; Piwuna Christopher Goson, MBBS, is a Senior Lecturer, Department of Psychiatry, College of Health Sciences; and Tanko Ishaya, PhD, is a Professor, Department of Computer Science; all at the University of Jos, Plateau State, Nigeria. Dipesh Bhattarai, PhD, is an Associate Lecturer, School of Medicine, Faculty of Health, Deakin University, Victoria, Australia. Khathutshelo Percy Mashige, PhD, is a Professor, Discipline of Optometry, African Vision Research Institute, Westville Campus, University of KwaZulu-Natal, Durban, South Africa. Richard Oloruntoba, PhD, is an Associate Professor, Supply Chain Management, School of Management and Marketing, Faculty of Business and Law, Curtin University, Bentley, Western Australia. Emmanuel Kwasi Abu, PhD, is Dean, Department of Optometry and Vision Science, School of Allied Health Sciences, University of Cape Coast, Ghana. Bernadine Ekpenyong, PhD, is Senior Lecturer, Department of Public Health, Faculty of Allied Medical Sciences, College of Medical Sciences, University of Calabar, Cross River State, Nigeria. Timothy G. Chikasirimobi, MSc, is a Master's Student, Masinde Muliro University of Science and Technology, Kakamega, Kenya. Godwin O. Ovenseri-Ogbomo, PhD, is an Assistant Professor, Department of Optometry, College of Applied Medical Sciences, Qassim University, Saudi Arabia, and Department of Optometry, Faculty of Life Sciences, University of Benin, Benin City, Nigeria. Raymond Langsi, MBBS, is Head, Health Division, University of Bamenda, Bambili, Cameroon. Deborah Donald Charwe, MSc, is Senior Research Nutritionist, Tanzania Food and Nutrition Center, Dar es Salaam, Tanzania. Obinna Nwaeze, MBBS, is a Practicing Physician, County Durham and Darlington, National Health Service Foundation, United Kingdom. Uchechukwu L. Osuagwu, Bernadine Ekpenyong, Godwin O. Ovenseri-Ogbomo, and Kingsley Emwinyore Agho are also Conjoint Members, Discipline of Optometry, African Vision Research Institute, Westville Campus, University of KwaZulu-Natal, Durban, South Africa.

**Keywords:** COVID-19, Infodemic, Misinformation, Public health preparedness/response, Epidemic management/response

## Abstract

Misinformation about coronavirus disease 2019 (COVID-19) is a significant threat to global public health because it can inadvertently exacerbate public health challenges by promoting spread of the disease. This study used a convenience sampling technique to examine factors associated with misinformation about COVID-19 in sub-Saharan Africa using an online cross-sectional survey. A link to the online self-administered questionnaire was distributed to 1,969 participants through social media platforms and the authors' email networks. Four false statements—informed by results from a pilot study—were included in the survey. The participants' responses were classified as “Agree,” “Neutral,” and “Disagree.” A multinomial logistic regression was used to examine associated factors. Among those who responded to the survey, 19.3% believed that COVID-19 was designed to reduce world population, 22.2% thought the ability to hold your breath for 10 seconds meant that you do not have COVID-19, 27.8% believed drinking hot water flushes down the virus, and 13.9% thought that COVID-19 had little effect on Blacks compared with Whites. An average of 33.7% were unsure whether the 4 false statements were true. Multivariate analysis revealed that those who thought COVID-19 was unlikely to continue in their countries reported higher odds of believing in these 4 false statements. Other significant factors associated with belief in misinformation were age (older adults), employment status (unemployed), gender (female), education (bachelor's degree), and knowledge about the main clinical symptoms of COVID-19. Strategies to reduce the spread of false information about COVID-19 and other future pandemics should target these subpopulations, especially those with limited education. This will also enhance compliance with public health measures to reduce spread of further outbreaks.

## Introduction

A great deal is still unknown about severe acute respiratory syndrome coronavirus 2 (SARS-CoV-2), the pathogen that causes coronavirus disease 2019 (COVID-19), which has resulted in a deluge of misinformation.^1,2^ The COVID-19 outbreak started in the Wuhan province of China in December 2019^3^ and spread rapidly across the world, as did conversations about the disease.^4^ Similar to other challenges, such as global warming, the impact of the COVID-19 pandemic depends on the actions of individuals, and, therefore, the quality of the information to which people are exposed. Notably, social media has been flooded with information regarding the origin and implications of the disease.^4,5^ Unfortunately, much of the information about COVID-19, its symptoms, transmission methods, and response mechanisms have been unreliable.^6-9^ As a result, audiences have been exposed to misinformation and misconceptions, an even disinformation, through propaganda and fake news, which need to be addressed.

Countries have been working to increase awareness and provide information to the public through various channels of communication (eg, radio, television advertisements, public health messages by prominent celebrities and national leaders, pamphlets and signboards at public places) about infection control measures and mode of infection; however, misinformation about COVID-19 remains. While some of the misinformation may be harmless, other misinformation could be dangerous and have implications for compliance with strategies designed to control the disease^9,10^ and may affect the development and implementation of possible treatments.^11^

Health authorities, including the World Health Organization (WHO) and the African Centres for Disease Control and Prevention, have included factual information as well as prevailing misinformation about COVID-19 on their websites to increase awareness.^7,12,13^ Additionally, some claims related to improving or boosting immunity against COVID-19 infection are being challenged.^7^ All of this has led to confusion among the general population.

Our interest is in sub-Saharan African countries where the pandemic arrived later and is home to over 1 billion people (14% of the world's population).^8^ The first confirmed case of COVID-19 in sub-Saharan Africa was reported in Nigeria on February 28, 2020. By April 1, 2020, 43 of the 46 sub-Saharan African countries had reported confirmed cases of COVID-19.^14^ Contrary to predictions of greater COVID-19 rates in the region,^15^ it remains one of the least affected regions in the world. This could be attributed to demonstrated solidarity and collective leadership. For example, African leaders adapted quickly to new preventive measures and the region has seen low international air traffic and has benefited from lessons learned from previous epidemics such as Ebola.^16^

With fragile healthcare systems, a catastrophic shortage of healthcare professionals,^17^ a drastic 75% reduction in medical commodities and supplies following border closures and restrictions on exports,^18^ and financial resource limitations, sub-Saharan Africa may still catch up with other regions of the world that have been more affected by COVID-19.^8^ The region needs to intensify its efforts to slow the spread of the pandemic by providing evidence-based information on the disease, using trusted channels^19,20^ to counter public misinformation, which will lay the foundation for sustained recovery.^21,22^ Identifying participatory ways of working will also be needed to put an end to the pandemic.

Studies have reported that belief in pseudoscience and myths about mental disorders was associated with a lower likelihood of health-seeking behavior in both the general population and medical professionals in India,^21^ and in a review of 66 articles, myths were a barrier to receiving hepatitis C treatment.^22^ Many parents in northern Nigeria avoided polio vaccinations for their children because of the myth that immunization causes infertility. Dispelling these types of myths may result in behavior change that could improve the health-seeking behavior of people.^21^ Additionally, recognizing and confronting misinformation head-on may serve to increase peoples' knowledge and their ability to accurately distinguish between and remember both mythical and factual information.^23^

In a study with 1,700 US adults, the authors found that nudging people to think about accuracy nearly tripled the level of true discernment in participants' subsequent sharing intentions,^24^ making it a simple way to tackle the sharing of false information. The purpose of our study was to analyze common misinformation about COVID-19 spreading across English-speaking countries in sub-Saharan Africa and to understand the underlying implications regarding the realities of social distancing and the use of face masks arising from specific myths. Findings from our study will provide people with reliable information using valid, evidence-based data to counter misinformation and misconceptions in sub-Saharan Africa related to the COVID-19 pandemic.

## Methods

This cross-sectional study used a convenience sampling technique to examine factors associated with misinformation about COVID-19 using an online survey.

### Survey Questionnaire

The survey tool for the COVID-19 knowledge questions was developed based on WHO guidelines for clinical and community management of COVID-19. The questionnaire was adapted with some modifications to identify the type of information and misinformation, obtain the respondent's attitude toward the mitigation practices, and analyze their potential compliance with strategies to control the spread of COVID-19 and their risk perception of contracting the disease.

We first conducted a pilot study to ensure the clarity and understanding of the questions asked and to determine the duration for completing the questionnaire. Ten participants from different English-speaking countries in sub-Saharan Africa took part in the pilot; they were not part of the research team and did not participate in the final survey. The pilot informed the selection of false information to include in the final survey. The final self-administered online survey (see Supplementary Annex, www.liebertpub.com/doi/suppl/10.1089/hs.2020.0202) consisted of 36 items divided into 4 sections: demographic characteristics, knowledge, perception, and practice. All questions about demographic characteristics were mandatory.

### Recruitment

The participants were sub-Saharan African nationals from different African countries—including Cameroon (distributed to only the English-speaking regions), Ghana, Kenya, Nigeria, South Africa, Tanzania, and Uganda—living either abroad or in their countries of origin. The survey was available only in English, and, therefore, participants were mostly from English-speaking countries. To be eligible for participation, participants had to be 18 years or older and able to provide online consent.

### Survey Distribution

A link to the survey was posted on social media platforms (Facebook and WhatsApp) commonly used by locals in the participating countries and was sent via email by the researchers to facilitate response. Participants were encouraged to share the survey link with their African networks. The survey was available online for 4 weeks (between April 18 and May 16, 2020) when most of the countries in sub-Saharan Africa were under mandatory lockdown and restriction of movement. Because it was not feasible to perform a nationwide community-based sample household survey during this period, data were obtained electronically via Survey Monkey. Only participants with access to the internet and who were on the respective social media platforms could participate.

The questionnaire included a brief overview of the context, purpose, and procedures of the survey; the nature of participation; privacy and confidentiality statements; and notes to be completed.^25^ To avoid multiple responses, participants were instructed not to complete the questionnaire a second time if they had already completed it. All eligible participants who completed the survey were included in the study.

To minimize bias, the survey used Likert scales with provisions for neutral responses, so the answers were not influenced. The participants did not receive any incentives and their responses were voluntary and anonymized. To test for the internal validity of the survey items, the Cronbach alpha (α) ranged from 0.70 to 0.74, indicating satisfactory consistency.

### Outcome Variables

There were 4 main outcome variables in this study, which were false statements about COVID-19. The false statements were selected due to their popularity among online users in sub-Saharan African countries, as informed by our pilot study. The 4 false statements were: (1) COVID-19 was designed to reduce world population, (2) COVID-19 has little effect on Blacks compared with Whites, (3) the ability to hold your breath for 10 seconds means you do not have COVID-19, and (4) drinking hot water flushes down the virus. Responses were designed using a 3-point Likert Scale.

### Covariates

The following variables were included in the analysis. Each question was scored on a 5-point Likert scale ranging from 0 (lowest) to 4 (highest).

*Demographic characteristics:* These variables included age, gender, marital status, place of residency, level of education, employment status, occupation, and religion.

*Knowledge of common symptoms of COVID-19:* These variables were included to account for shifting knowledge about the disease in the analysis. Questions included whether participants could identify common symptoms of COVID-19 (fever, dry cough, and fatigue—as listed by WHO) as the main clinical symptoms of the disease^26^ and how they differ from common cold symptoms.

*Attitude toward COVID-19:* These variables were included because they influence people's actions to reduce the spread of infection. Survey questions inquired about the practice of self-isolation, home quarantine, and the number of people living together in the household.

*Compliance with precautionary public health measures:* These variables were included to understand compliance with precautionary measures in place to mitigate the spread of COVID-19 during lockdown. Survey questions asked whether the participants had gone to any crowded place including religious events, if they wore a mask when leaving home, and if, in recent days, they had been washing their hands with soap for at least 20 seconds each time or using hand sanitizers.

*COVID-19 risk perception*: These variables were included because individuals who perceive risk are more likely to reduce the spread of the virus. Survey questions about risk perception included whether respondents thought they were at risk of becoming infected, at risk of dying from the infection, how worried they were about COVID-19, and how likely they thought it was that COVID-19 would continue in their country.

### Statistical Analysis

Data cleaning, sorting, and processing were carried out before the analysis. Tabulation was used to determine the prevalence (and 95% confidence intervals [CI]) of beliefs in the 4 false statements about COVID-19. Responses were categorized as “Agree” (2), “Neutral” (1), or “Disagree” (0). Over one-third of responses were “Neutral,” which was analyzed separately, as adding this category to either the “Agree” or “Disagree” category would bias the study findings^27^ and their policy implications. Univariate and multivariate multinomial logistic regression were used to determine associated factors, after controlling for individual confounding variables.

Multinomial logistic regression using a manual stepwise backward approach was used to identify the factors associated with the 4 false statements about COVID-19. The results were presented as unadjusted odds ratios and adjusted odds ratios (AOR) with corresponding 95% CI. All variables with a statistical significance of *P* ≤ .05 were retained in the final model. The AOR and analyses were performed using Stata version 14.1 (Stata Corp, College Station, Texas).

### Ethical Approval

Ethical approval for the study was obtained from the Human Research Ethics Committee of the Cross River State Ministry of Health (CRSMOH/HRP/HREC/2020/117). The study adhered to the tenets of the Declaration of Helsinki regarding research involving human subjects, and informed consent was obtained from all participants before completing the survey. Participants were required to answer “yes” or “no” to the consent question during survey completion to indicate their willingness to participate in this study. All those who agreed to voluntarily participate in the survey were included in the study. The confidentiality of participants was assured in that no identifying information was obtained from participants.

## Results

Overall, 1,969 participants (55.2% male, 44.8% female) responded to the survey. Respondents' age categories were 18 to 28 years (39.0%), 29 to 38 years (26.7%), 39 to 48 years (22.2%), and 49 years or older (12.1%). Over half (n = 1,108, 56.3%) of respondents were from West Africa, whereas a tenth (n = 209, 10.6%) were from East Africa. A majority (79.2%) of respondents had at least a bachelor's degree and 20.8% had only a primary (basic) or secondary level of education. The majority of respondents (>81%) correctly identified fever, dry cough, and fatigue as the main clinical symptoms of COVID-19; their responses were split (50.7% versus 49.3%) on whether people with COVID-19 were more or less likely to experience the symptoms of a common cold. Table 1 shows a detailed summary of participants' demographic characteristics.

**Table 1.** Demographic Characteristics of Survey Respondents

Variables	n (%)
*Demography*	
Region	
West Africa	1,108 (56.3)
East Africa	209 (10.6)
Central Africa	251 (12.7)
Southern Africa	401 (20.4)
Place of residence	
Locally (Africa)	1855 (92.5)
Diaspora	150 (7.5)
Age category (years)	
18-28	775 (39.0)
29-38	530 (26.7)
39-48	441 (22.2)
49+	242 (12.1)
Sex	
Male	1099 (55.2)
Female	892 (44.8)
Marital status	
Married	879 (44.1)
Unmarried	1116 (55.9)
Highest level of education	
Postgraduate degree (master's/PhD)	642 (32.2)
Bachelor's degree	939 (47.0)
Primary/secondary	416 (20.8)
Employment status	
Employed	1321 (66.0)
Unemployed	679 (34.0)
Religion	
Christian	1763 (88.4)
Others	232 (11.6)
Occupation	
Nonhealthcare sector	1471 (77.3)
Healthcare sector	433 (22.7)
Number of people living together in 1 household	
<3 people	506 (28.8)
4-6 people	908 (51.7)
6+ people	341 (19.4)
*Knowledge of symptoms of COVID-19*	
Fever	
No	36 (2.0)
Yes	1776 (98.0)
Fatigue	
No	324 (18.7)
Yes	1408 (81.3)
Dry cough	
No	324 (2.8)
Yes	1759 (97.2)
Sore throat	
No	215 (12.0)
Yes	1,580 (88.0)
Unlike cold symptoms	
No	907 (49.3)
Yes	931 (50.7)
*Attitude toward COVID-19*	
Self-isolation	
No	1237 (66.7)
Yes	564 (31.3)
Home quarantined due to COVID-19	
No	1091 (60.7)
Yes	707 (39.3)
*Compliance during COVID-19 lockdown*	
Gone to crowded places including religious events	
No	1097 (54.0)
Yes	935 (46.0)
Wore mask when going out	
No	485 (23.9)
Yes	1547 (76.1)
Practiced regular handwashing	
No	762 (37.5)
Yes	1270 (62.5)
*COVID-19 risk perception*	
Risk of becoming infected	
High	669 (37.2)
Low	1128 (62.8)
Risk of becoming severely infected	
High	466 (25.9)
Low	1333 (74.1)
Risk of dying from the infection	
High	349 (19.5)
Low	1445 (80.6)
How worried are you because of COVID-19?	
Worried	1037 (57.5)
Not worried	766 (42.5)
How likely do you think COVID-19 will continue in your country?	
Very likely	1152 (64.0)
Not very likely	649 (36.0)
Concern for self and family if COVID-19 continues	
Concerned	1667 (94.2)
Not concerned	102 (5.8)

### Prevalence of Belief in Misinformation About COVID-19

**Figure 1.**
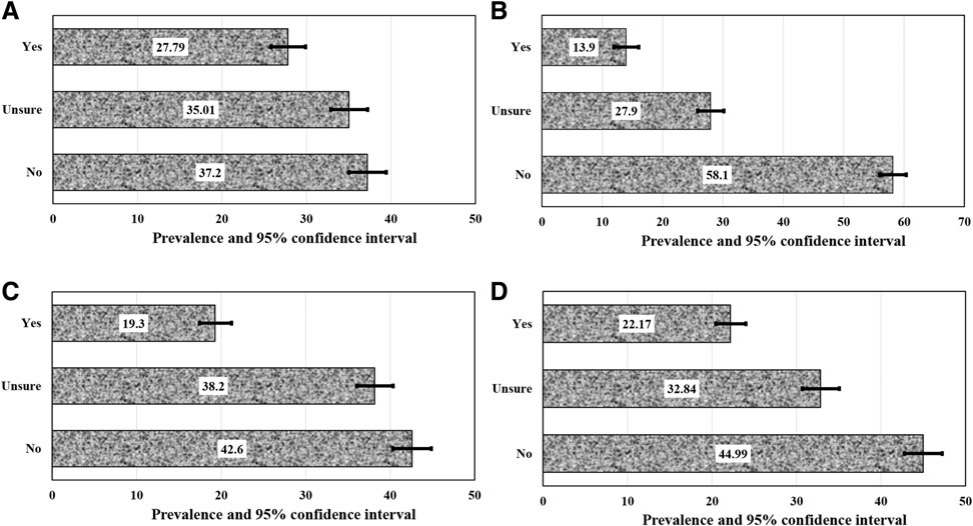
Prevalence of belief in false statements related to COVID-19: (a) drinking hot water flushes down the virus; (b), COVID-19 has little effect on Blacks compared with Whites; (c) COVID-19 was designed to reduce world population; and (d) the ability to hold your breath for 10 seconds means you don't have COVID-19.

Figure 1 presents the prevalence of belief in the following 4 false statements about COVID-19: (1) drinking hot water flushes down the virus, (2) COVID-19 has little effect on Blacks compared with Whites, (3) COVID-19 was designed to reduce world population, and (4) the ability to hold your breath for 10 seconds means you do not have COVID-19. The figure shows that 27.8% of respondents thought that drinking hot water flushes down the virus, followed by 22.2% who thought the ability to hold your breath for 10 seconds means you do not have COVID-19. About one-fifth (19.3%) of respondents believed that COVID-19 was designed to reduce world population, and more than one-third (38.2%) of respondents were unsure about the false misinformation. On whether respondents thought that COVID-19 has less of an effect on Blacks than Whites, 13.9% agreed and 27.9% were neutral.

### Beliefs in 4 False Statements About COVID-19

The unadjusted odd ratios and 95% CI of beliefs in the 4 false statements about COVID-19 are presented in the Supplementary Tables (www.liebertpub.com/doi/suppl/10.1089/hs.2020.0202). Supplementary Table 1 shows that age (39 to 48 years), marital status (not married, which included single, divorced, separated, or widowed), religion (others, including Muslims and African Traditionalists), level of education (bachelor's degree), noncompliance with the public health measures (going to crowded places including religious events and use of face masks when going outside), level of perceived risk (not worried about COVID-19, and perceived low risk of becoming severely infected), and level of perceived likelihood that COVID-19 would continue in their country were significantly associated with the belief that drinking hot water flushes down the virus.

The variables associated with the belief that COVID-19 has less of an effect on Blacks than Whites (Supplementary Table 2) included subregion of residency (East Africa), employment status (unemployed), marital status (not married), religion (non-Christians), education level (bachelor's degree), noncompliance with the public health measures (gone to crowded places including religious events), and level of perceived risk of contracting COVID-19 infection (not worried about COVID-19). In addition to these variables, gender (female) played a significant role in the false belief that COVID-19 is designed to reduce world population (Supplementary Table 3). In regard to the false belief that the ability to hold your breath for 10 seconds means you do not have COVID-19 (Supplementary Table 4), subregion of residency (Central Africa) and the level of perceived risk of contracting COVID-19 were significant variables. These variables were further analyzed after adjusting for potential confounders.

**Table 2.** Multinomial Logistic Regression of Factors Associated with Misinformation Related to COVID-19

	Neutral		Agree	
Variables	AOR (95% CI)	P Value	AOR (95% CI)	P Value
***a. Factors associated with belief in false statement 1: Drinking hot water flushes down the virus***				
*Demography*				
Age category (years)				
18-28	1.00		1.00	
29-38	1.42 (0.99-2.03)	.056	1.86 (1.25-2.77)	.002
39-48	**2.22 (1.47-3.36)**	**<.001**	**3.61 (2.30-5.67)**	**<.001**
49+	**1.86 (1.16-3.00)**	**.011**	**3.16 (1.90-5.26)**	**<.001**
Employment status				
Employed	1.00		1.00	
Unemployed	**1.62 (1.16-2.27)**	**.005**	**1.72 (1.19-2.50)**	**.004**
Religion				
Christian	1.00		1.00	
Others	0.64 (0.44-0.93)	.020	0.67 (0.45-1.01)	.053
Highest level of education				
Postgraduate degree (master's/PhD)	1.00		1.00	
Bachelor's degree	**1.83 (1.36-2.45)**	**<.001**	**1.84 (1.35-2.51)**	**<.001**
Primary/secondary	0.93 (0.58-1.49)	.771	1.36 (0.83-2.22)	.217
*Knowledge of symptoms of COVID-19*				
Fatigue				
No	1.00		1.00	
Yes	0.88 (0.64-1.19)	.404	0.69 (0.50-0.96)	.025
Sore throat				
No	1.00		1.00	
Yes	**1.60 (1.12-2.30)**	**.010**	**1.71 (1.15-2.54)**	**.008**
*Compliance during COVID-19 lockdown*				
Gone to crowded place including religious events				
No	1.00		1.00	
Yes	1.25 (0.98-1.60)	.069	1.36 (1.05-1.77)	.020
*COVID-19 risk perception*				
If COVID-19 continues, how concerned would you be that you or family would be directly affected?				
Concerned	1.00		1.00	
Not concerned	1.05 (0.64-1.73)	.836	0.69 (0.39-1.24)	.215
How likely do you think COVID-19 will continue in your country?				
Likely	1.00		1.00	
Not likely	**1.74 (1.35-2.24)**	**<.001**	**1.90 (1.45-2.48)**	**<.001**
***b. Factors associated with belief in false statement 2: COVID-19 has little effect on Blacks compared with Whites***				
*Demography*				
Subregion				
Southern Africa	1.00		1.00	
Central Africa	1.36 (0.93-1.97)	.111	1.37 (0.85-2.22)	.201
East Africa	1.30 (0.90-1.88)	.165	2.07 (1.36-3.15)	.001
West Africa	1.31 (0.98-1.73)	.065	0.95 (0.63-1.42)	.793
Religion				
Christian	1.00		1.00	
Others	0.63 (0.43-0.93)	.020	0.61 (0.36-1.03)	.065
Highest level of education				
Postgraduate degree (master's/PhD)	1.00		1.00	
Bachelor's degree	1.43 (1.11-1.84)	.006	1.34 (0.96-1.87)	.088
Primary/secondary	1.07 (0.72-1.59)	.731	1.14 (0.69-1.89)	.602
*Knowledge of symptoms of COVID-19*				
Fever				
No	1.00		1.00	
Yes	0.43 (0.20-0.92)	.030	0.41 (0.16-1.05)	.064
*Compliance during COVID-19 lockdown*				
Gone to crowded place including religious events				
No	1.00		1.00	
Yes	1.28 (1.01-1.61)	.042	1.35 (0.99-1.82)	.053
Handwashing/used hand sanitizer				
No	1.00		1.00	
Yes	**0.77 (0.60-0.98)**	**.035**	**0.62 (0.45-0.84)**	**.002**
*COVID-19 risk perception*				
How likely do you think COVID-19 will continue in your country?				
Likely	1.00		1.00	
Not likely	**1.88 (1.48-2.38)**	**<.001**	**2.53 (1.87-3.42)**	**<.001**
***c. Factors associated with belief in false statement 3: COVID-19 was designed to reduce world population***				
*Demography*				
Age category (years)				
18-28	1.00		1.00	
29-38	1.13 (0.81-1.57)	.475	0.63 (0.42-0.94)	.024
39-48	0.97 (0.67-1.42)	.882	0.48 (0.30-0.79)	.004
49+	0.86 (0.56-1.32)	.489	0.43 (0.24-0.76)	.004
Gender				
Male	1.00		1.00	
Female	1.11 (0.89-1.38)	.368	1.54 (1.17-2.02)	.002
Subregion				
Southern Africa	1.00		1.00	
Central Africa	1.16 (0.80-1.68)	.440	1.45 (0.93-2.27)	.104
East Africa	**1.55 (1.08-2.21)**	**.017**	**1.68 (1.10-2.56)**	**.016**
West Africa	0.99 (0.75-1.31)	.964	0.85 (0.60-1.21)	.375
Employment status				
Employed	1.00		1.00	
Unemployed	**1.54 (1.12-2.11)**	**.008**	**1.85 (1.28-2.68)**	**.001**
Highest level of education				
Postgraduate degree (master's/PhD)	1.00		1.00	
Bachelor's degree	**1.43 (1.10-1.85)**	**.007**	**1.69 (1.17-2.43)**	**.005**
Primary/secondary	1.07 (0.69-1.64)	.771	1.30 (0.78-2.19)	.317
*Compliance during COVID-19 lockdown*				
Gone to crowded place including religious events				
No	1.00		1.00	
Yes	1.32 (1.06-1.66)	.015	1.18 (0.89-1.56)	.259
*COVID-19 risk perception*				
How likely do you think COVID-19 will continue in your country?				
Likely	1.00		1.00	
Not likely	**2.00 (1.59-2.51)**	**<.001**	**1.55 (1.16-2.07)**	**.003**
***d. Factors associated with belief in false statement 4: The ability to hold your breath for 10 seconds means you don't have COVID-19***				
*Demography*				
Subregion				
Southern Africa	1.00		1.00	
Central Africa	1.05 (0.72-1.52)	.803	0.59 (0.37-0.94)	.026
East Africa	1.20 (0.84-1.72)	.320	1.09 (0.74-1.62)	.655
West Africa	0.94 (0.70-1.27)	.702	0.72 (0.52-0.99)	.049
Number of people living together in 1 household				
<3 people	1.00		1.00	
4-6 people	1.34 (1.01-1.76)	.040	1.23 (0.91-1.66)	.186
6+	1.18 (0.84-1.65)	.353	0.82 (0.56-1.23)	.339
*Knowledge of symptoms of COVID-19*				
Unlike cold symptoms				
No	1.00		1.00	
Yes	0.77 (0.61-0.97)	.026	0.85 (0.65-1.11)	.226
*COVID-19 risk perception*				
How worried are you because of COVID-19?				
Worried	1.00		1.00	
Not worried	0.86 (0.68-1.10)	.228	0.74 (0.56-0.97)	.027
How likely do you think COVID-19 will continue in your country?				
Likely	1.00		1.00	
Not likely	**2.09 (1.63-2.68)**	**<.001**	**1.75 (1.32-2.31)**	**<.001**

Note: Variables set in bold are common factors associated with the belief or uncertainty in the false statements about COVID-19.

Abbreviations: AOR, adjusted odds ratio; CI, confidence interval.

### Variables Associated with Misinformation About COVID-19

Analysis of the variables associated with belief in the misinformation is presented in the section that follows. Table 2 shows the variables associated with the 4 false statements about COVID-19.

#### False Statement 1: Drinking Hot Water Flushes Down the Virus

Older respondents, those who were unemployed, and those who had at least a bachelor's degree were more likely to believe that drinking hot water flushes down the COVID-19 virus. The odds of believing that drinking hot water flushes down the virus was lower among participants who correctly identified fatigue (AOR = 0.69; 95% CI, 0.50 to 0.96) and higher among those who wrongly identified sore throat (AOR = 1.71; 95% CI, 1.15 to 2.54) as one of the main clinical symptoms of the disease at the time of this study. Noncompliance with the precautionary health measure urging people to avoid attending crowded places, including religious events, increased the odds of believing that drinking hot water flushes down the virus (AOR = 1.36; 95% CI, 1.05 to 1.77). Those who perceived that COVID-19 is not likely to continue in their countries were about 2 times more likely to agree with this false statement compared with other respondents (AOR = 1.90; 95% CI, 1.45 to 2.48). Similar trends in significance were observed among those who were “neutral.” Respondents were more likely to be neutral to this misinformation if they were older, unemployed, non-Christians, held at least a bachelor's degree, visited crowded places during the lockdown, and thought that COVID-19 was not likely to continue in their countries after the lockdown.

#### False Statement 2: COVID-19 Has Little Effect on Blacks Compared with Whites

East African respondents were more likely than Southern African respondents to agree with the misinformation that COVID-19 has little effect on Blacks compared with Whites (AOR = 2.07; 95% CI, 1.36 to 3.15). The respondents who did not wash their hands or did not use hand sanitizer were more likely to agree with this misinformation. Similarly, the respondents who perceived that COVID-19 was not likely to continue in their country (AOR = 2.53; 95% CI, 1.87 to 3.42) had a higher likelihood of reporting that Blacks are less affected. Similarly, a significant proportion of respondents who held at least a bachelor's degree (AOR = 1.43; 95% CI, 1.11 to 1.84), non-Christians, respondents who visited crowded places during the lockdown, and those who thought COVID-19 will not continue in their respective countries were more likely to be neutral on the belief that COVID-19 has little effect on Blacks compared with Whites. Respondents who were unsure of the common clinical symptoms of the disease had lower odds of belief in this misinformation.

#### False Statement 3: COVID-19 Was Designed to Reduce World Population

Female respondents and those with lower education were more likely to agree with the false statement that COVID-19 was designed to reduce world population. There were significant associations between belief in this misinformation and residing in the East African region. Respondents who thought there was a low likelihood of COVID-19 continuing in their countries were more likely to agree with the statement (AOR = 1.55; 95% CI, 1.16 to 2.07). A similar trend of significance was found in the “neutral” group. East Africans, those who were unemployed (AOR = 1.54; 95% CI, 1.12 to 2.11), visited crowded places or religious events (AOR = 1.32; 95% CI, 1.06 to 1.66), and those who thought the likelihood of COVID-19 continuing in their country was low were more likely to respond neutrally to the false statement that COVID-19 was designed to reduce world population.

#### False Statement 4: The Ability to Hold Your Breath for 10 Seconds Means You Do Not Have COVID-19

Central and West African respondents were less likely to believe that the ability to hold your breath for 10 seconds means you do not have COVID-19 compared with Southern Africans (AOR = 0.59; 95% CI, 0.37 to 0.94; AOR = 0.72, 95% CI, 0.52 to 0.99). Similarly, respondents who were worried about contracting COVID-19 or thought there was a low likelihood of COVID-19 continuing in their countries were more likely to agree with or be neutral to this misinformation. The association between household factors (living with 4 to 6 people) and respondents who neither agreed nor disagreed with the false statement that holding your breath for 10 seconds means you do not have COVID-19 was also significant (AOR = 1.34; 95% CI, 1.01 to 1.76). Respondents who thought there was a low likelihood of COVID-19 continuing in their respective countries were about 2 times more likely to be neutral to this misinformation, compared with those who thought the disease was more likely to continue in their countries. Knowledge of the common clinical symptoms of COVID-19 was associated with a reduced risk, particularly among those who were neutral to this misinformation.

## Discussion

This study assessed 4 common false statements related to COVID-19 and their determinants across English-speaking countries in sub-Saharan Africa. We found that about 1 in every 5 (21%) respondents believed that drinking hot water flushes down the virus and that the ability to hold your breath for 10 seconds means you do not have COVID-19. Some (14%) participants also believed that COVID-19 has little effect on Blacks compared with Whites, and that the disease was designed to reduce world population. In addition, a reasonable proportion (34%) of the participants were unsure whether the 4 false statements were true. The common characteristics associated with belief in the misinformation were age (older adults), gender (female), place of residency (East Africa), education (bachelor's degree), and employment status (unemployed). Additionally, those who were knowledgeable about the common clinical symptoms of COVID-19 had lower odds of belief in the misinformation. Participants who agreed with any of the false statements demonstrated low-risk perception for contracting the infection and poor attitude toward WHO precautionary public health measures put in place to contain the spread of the infection in their countries.

The study showed that belief in misinformation about COVID-19 was predominant among the older respondents in sub-Saharan Africa who have the highest risk of developing severe complications from COVID-19.^28^ This finding is corroborated by a recent study, which found that older adults are up to 7 times more likely to share misinformation than their younger counterparts.^29^ To more effectively target the spread of misinformation among older adults, there is need to look more closely at interpersonal relationships and digital literacy. In addition to being less likely to use social platforms than younger generations, older adults tend to have fewer people in their social spheres and tend to have more trust in the people they do know.^29^

Belief in misinformation about COVID-19 has been associated with a poor attitude toward public health precautionary measures, which ultimately can lead to increased COVID-19 infections,^30,31^ as well as psychosocial, economic, and ethical consequences.^32^ Respondents who were unemployed were more likely to believe in misinformation about COVID-19, and as shown in a previous study,^33^ individuals with low incomes had a higher risk of mortality due to COVID-19 infection. Public health efforts to reduce COVID-19 should, therefore, integrate targeted interventions to specific population subgroups to ensure their effectiveness in a high-risk population. For instance, accountable mass media that disseminates socially and culturally acceptable preventives measures of COVD-19 can not only mitigate misinformation but also reduce the mental health impacts of COVID-19 among the older population.^34^ Health communication that fosters wellbeing and addresses basic psychological needs has the potential to cut through the infodemic and promote effective and sustainable behavior change during a pandemic.^35^

The finding that respondents from East Africa were more likely to agree with most of the misinformation about COVID-19 was not surprising. Despite imposing curfews, partial and full lockdowns, and enforcing physical distancing in Tanzania, President John Magufuli still believed that COVID-19 was the work of the devil. During the lockdown, he encouraged people to attend public worship in churches and mosques, insisting that “prayer can defeat coronavirus disease.”^36^ On the other hand, Kenya, the closest neighbor to Tanzania, introduced a media and information literacy policy in their national school curriculum and recently took specific actions to apply these policies to combat COVID-19 disinformation. It is anticipated that media and information literacy policies will help create a media-literate population with the capacity and skills to access quality information, which they need to make informed decisions within the new media and information environment.^37^ Although there is no evidence on the impact of introducing such a media and information literacy policy on misinformation spread during the COVID-19 pandemic, the Kenyan government—with support from the United Nations Educational, Scientific, and Cultural Organization—held several training sessions targeting media practitioners, regulators, and stakeholders during the pandemic. Training was conducted to improve the quality of journalism and provide trusted sources of information to strengthen media and information literacy.^37^

Noncompliance with public health measures to mitigate the spread of COVID-19, such as avoiding crowds and practicing good hand hygiene, was associated with belief in misinformation about the pandemic. During the current COVID-19 crisis, in the absence of vaccines for everyone, public health measures to avoid large gatherings, keep good hand hygiene, and use face masks are the main directives in place to prevent and control widespread outbreaks. Public health initiatives to reduce misinformation among the general population can prevent the violation of measures put in place to mitigate the spread of the pandemic. Using evidence-based health literacy strategies, such as plain language, social media can be an effective tool to promote health literacy among any targeted population.^38^ Mass campaigns using social media platforms with clear messages from public health and local authorities to encourage social distancing and use of face masks can help prevent the uncontrolled spread of the virus.^39^ While it is important to provide correct information about COVID-19, it is even more vital that such information is provided using trusted sources such as government-owned broadcast media,^40^ celebrities,^19^ and trained community health advisors.^20^

The belief that COVID-19 was deliberately developed and spread is common not only in lower-income countries^41^ but also in higher-income countries including the United States and Australia.^42,43^ A study conducted in the United States showed that around one-third of respondents agreed with this misinformation.^42^ More than half of the participants in our study either agreed with or were neutral about a similar false statement that COVID-19 was designed to reduce world population. East Africans, female respondents, people who were unemployed, and people with at least a bachelor's degree were more likely to agree or be neutral about this misinformation.

This study has some limitations. The survey was conducted online, and, therefore, it may not be representative of the opinion of those living in rural areas where internet access is relatively low.^44^ Because respondents were self-selected, there was no way to differentiate characteristics of respondents and nonrespondents. It was also difficult to prevent multiple responses from 1 person,^45^ although respondents were instructed not to complete the survey more than once. Although the study may not have captured the beliefs of older people who are less likely to use internet compared with younger people,^45^ an online survey was the only reliable means to disseminate information at the time of the study and provided an innovative way to collect real-time data. Studies have found an increase in use of the internet among the general population during the pandemic,^46^ but it is unlikely to have significantly impacted the results presented. In addition, the low cost and overall availability of the survey to a large number of people at any time of the day, in addition to being able to process the data in real-time, made the use of an online survey a preferred data collection tool. The survey was available only in English, and, therefore, some respondents from French-speaking countries did not participate. Participation of respondents from East Africa may have been affected by the lockdown, as residents of Kenya and Tanzania were asked to refrain from giving out information regarding the pandemic, which may have resulted in a wide variation in the response rate by region. Another limitation of this study was the use of a “neutral” option in the questionnaire without specifically defining what the selection of this option means. Nadler et al^27^ found that the selection of the neutral option may be measuring different attitudes and that participants tend to overuse this option in questionnaires. They also noted that providing respondents with the neutral option would minimize response bias. There were no incentives given to participants in this study, and no assistance was sought from online companies during the distribution of the survey, which may have affected the reach of the survey. Lastly, this study is limited by the fact that it did not examine the changing symptom profiles and knowledge of COVID-19, which has evolved over time. However, future research on misinformation should consider the changing profile in knowledge of the disease and symptoms, and how that affects people's beliefs in misinformation. Despite these limitations, this is the first study to provide robust and comprehensive evidence of beliefs in common misinformation about COVID-19 in English-speaking countries in sub-Saharan Africa. Previous studies describing other misinformation do not explore how other factors are associated with beliefs in misinformation, and they lack the robust statistical analysis to explore how such misinformation and other variables are related.^47^ In addition, efforts were made to minimize bias in this online survey.

## Conclusion

Misinformation about COVID-19 is prevalent among East Africans and is associated with age (older adults), gender (female), and employment status (unemployed). There is a clear association between susceptibility to misinformation and having knowledge about the clinical symptoms of COVID-19, low risk perception of becoming infected, and noncompliance with public health measures. The results of our study highlight a need to combat the COVID-19 infodemic across English-speaking countries in sub-Saharan Africa. Strengthening the health literacy of the general population in the participating sub-Saharan African countries is an effective approach to protect people from misinformation. Interventions to increase compliance and improve critical thinking and trust in science will be a promising avenue for future research. In addition, teaching public health literacy, such as how to verify the source of information and other useful methods, are necessary to combat misinformation. Sub-Saharan African countries will benefit from engaging with nongovernmental organizations at the community level, and countries could go even further to convince people by providing accurate information in local languages. A strategy (or combination of strategies) that ensures effective health communication to improve public knowledge or change health behavior should be associated with a measurable effect on health outcomes.

## Supplementary Material

Supplemental data

Supplemental data

Supplemental data

Supplemental data

Supplemental data
