# Supplementary material for: Misinformation About COVID-19 in Sub-Saharan Africa: Evidence from a Cross-Sectional Survey
Source: Health Secur. 2021 Feb 18;19(1):44–56. doi: 10.1089/hs.2020.0202 (PMC9347271; doi:10.1089/hs.2020.0202)
Supplement: Supplemental data [file Supp_Data.docx]

Supplementary Annex. Sample of survey tool used in the study

1. **CONSENT**

I willingly agree to participate in this survey because I am interested in contributing to the knowledge and perceptions on Coronavirus disease (COVID-19) Pandemic. I understand that there are no forms of payments or reward associated with my participation.

UNDERSTOOD, AGREE AND INTERESTED

NOT UNDERSTOOD, DISAGREE AND NOT-INTERESTED

1. Country of origin
2. Country of residence
3. Province/State/County
4. Gender

MALE

FEMALE

OTHERS

1. Age (Years)
2. Marital Status

SINGLE

MARRIED

SEOARATED/DIVORCED

WIDOW/WIDOWER

1. Religion

MUSLIM

CHRISTIAN

AFRICAN TRADITIONALIST

OTHERS

1. Highest level of education

PRIMARY SCHOOL

HIGH/SECONDARY SCHOOL

POLYTHECNIC/DIPLOMA

UNIVERSITY DEGREE (Bachelors/Professional)

POSTGRADUATE DEGREE (Masters/PhD)

1. Employment Status

SELF EMPLOYED

EMPLOYED

UNEMPLOYED

STUDENT/NON-STUDENT

1. Occupation
2. If you live with family/friends, how many of you live together?

**General KNOWLEDGE of COVID-19**

1. Do you think Coronavirus disease (COVID-19) has little effect(s) on Blacks than on Whites?

YES

NO

NOT SURE

1. Do you think Coronavirus disease (COVID-19) was designed to reduce world population?

YES

NO

NOT SURE

1. The main clinical symptoms of Coronavirus disease (COVID-19) are: (Type "YES" or "NO" to the suggested options as applicable)

FEVER

FATIGUE

DRY COUGH

SORE THROAT

1. Unlike the common cold, stuffy nose, runny nose, and sneezing are less common in persons infected with the COVID-19 virus.

TRUE

FALSE

NOT SURE

1. There currently is no effective cure for COVID-2019, but early symptomatic and supportive treatment can help most patients recover from the infection

TRUE

FALSE

NOT SURE

1. It is not necessary for children and young adults to take measures to prevent the infection by the COVID-19 virus.

TRUE

FALSE

NOT SURE

1. COVID-19 individuals cannot spread the virus to anyone if there's no fever.

TRUE

FALSE

NOT SURE

1. The COVID-19 virus spreads via respiratory droplets of infected individuals

TRUE

FALSE

NOT SURE

1. To prevent getting infected by Coronavirus disease (COVID-19), individuals should avoid going to crowded places such as train stations, religious gatherings, and avoid taking public transportation

TRUE

FALSE

NOT SURE

1. Isolation and treatment of people who are infected with the Coronavirus disease (COVID-19) virus are effective ways to reduce the spread of the virus. The observation period is usually 14 days

TRUE

FALSE

NOT SURE

1. Not all persons with COVID-2019 will develop to severe cases. Only those who are elderly, have chronic illnesses, and are obese are more likely to be severe cases.

TRUE

FALSE

NOT SURE

**PERCEPTION OF RISK OF INFECTION**

1. Risk of becoming infected.

VERY HIGH

HIGH

LOW

VERY LOW

UNLIKELY

1. Risk of becoming severely infected

VERY HIGH

HIGH

LOW

VERY LOW

UNLIKELY

1. Risk of dying from the infection

VERY HIGH

HIGH

LOW

VERY LOW

UNLIKELY

1. How worried are you because of COVID-19?

A GREAT DEAL

A LOT

A MODERATE AMOUNT

A LITTLE

NONE AT ALL

1. Do you think that if you are able to hold your breath for 10 seconds, it's a sign that you don't have COVID-19?

YES

NO

NOT SURE

1. If you drink hot water, it flushes down the virus

STRONGLY AGREE

AGREE

NEITHER AGREE, NOR DISAGREE

DISAGREE

STRONGLY DISAGREE

1. How likely do you think Coronavirus disease (COVID-19) will continue in your country?

VERY LIKELY

LIKELY

NEITHER LIKELY, NOR UNLIKELY

UNLIKELY

VERY UNLIKELY

1. If Coronavirus disease (COVID-19) continues in your country, how concerned would you be that you or your family would be directly affected?

EXTREMELY CONCERNED

CONCERNED

NEITHER CONCERNED, NOR UNCONCERNED

UNCONCERNED

EXTREMELY UNCONCERNED

**PRACTICE REGARDING COVID-19**

1. In recent days, have you gone to any crowded place including religious events?

ALWAYS

SOMETIMES

RARELY

NOT AT ALL

NOT SURE

1. In recent days, have you worn a mask when leaving home?

ALWAYS

SOMETIMES

RARELY

NOT AT ALL

NOT SURE

1. In recent days, have you been washing your hands with soap and running water for at least 20 seconds each time?

ALWAYS

SOMETIMES

RARELY

NOT AT ALL

NOT SURE

1. Are you currently or have you been in (domestic/home) quarantine because of COVID-19?

YES

NO

1. Are you currently or have you been in self-isolation because of COVID-19?

YES

NO

**THANK YOU FOR TAKING OUR SURVEY**

(Source: Revised and Adopted from WHO, 2020)
