# Supplementary material for: Misinformation About COVID-19 in Sub-Saharan Africa: Evidence from a Cross-Sectional Survey
Source: Health Secur. 2021 Feb 18;19(1):44–56. doi: 10.1089/hs.2020.0202 (PMC9347271; doi:10.1089/hs.2020.0202)
Supplement: Supplemental data [file Supp_Table1.docx]

Supplementary Table 1. Univariate analysis of factors associated with the misconception that drinking hot water flushes down COVID-19

| **Variables** | **Neutral** | | **Agree** | |
| --- | --- | --- | --- | --- |
|  | **Unadjusted OR [95% CI]** | **P-value** | **Unadjusted OR [95% CI]** | **P-value** |
| **Age (years)** |  |  |  |  |
| 18-28 | 1.00 |  | 1.00 |  |
| 29-38 | 1.01 [0.77, 1.32] | 0.968 | 1.18 [0.88, 1.59] | 0.260 |
| 39-48 | 1.28 [0.96, 1.73] | 0.097 | 1.79 [1.31, 2.44] | <0.001 |
| 49+ | 1.06 [0.74, 1.52] | 0.744 | 1.41 [0.97, 2.06] | 0.072 |
| **Gender** |  |  |  |  |
| Male | 1.00 |  | 1.00 |  |
| Female | 1.08 [0.87, 1.34] | 0.501 | 1.16 [0.92, 1.47] | 0.202 |
| **Sub-region** |  |  |  |  |
| Southern Africa | 1.00 |  | 1.00 |  |
| Central Africa | 1.00 [0.68, 1.47] | 0.988 | 1.31 [0.90, 1.92] | 0.159 |
| East Africa | 1.48 [1.04, 2.09] | 0.028 | 1.24 [0.85, 1.80] | 0.266 |
| West Africa | 1.09 [0.83, 1.43] | 0.546 | 0.79 [0.58, 1.08] | 0.143 |
| **Region of residence** |  |  |  |  |
| Africa | 1.00 |  | 1.00 |  |
| Diaspora | 1.07 [0.70, 1.62] | 0.760 | 1.03 [0.66, 1.61] | 0.885 |
| **Employment status** |  |  |  |  |
| Employed | 1.00 |  | 1.00 |  |
| Unemployed | 1.10 [0.87, 1.38] | 0.427 | 0.94 [0.74, 1.21] | 0.634 |
| **Marital Status** |  |  |  |  |
| Married | 1.00 |  | 1.00 |  |
| Not married | 1.01 [0.81, 1.26] | 0.905 | 0.76 [0.61, 0.96] | 0.023 |
| **Religion** |  |  |  |  |
| Christianity | 1.00 |  | 1.00 |  |
| Others | 0.59 [0.42, 0.83] | 0.002 | 0.61 [0.42, 0.87] | 0.007 |
| **Highest level of Education** |  |  |  |  |
| Postgraduate Degree (Masters /PhD) | 1.00 |  | 1.00 |  |
| Bachelor’s degree | 1.60 [1.26, 2.03] | <0.001 | 1.36 [1.06, 1.76] | 0.017 |
| Secondary/Primary | 0.79 [0.55, 1.15] | 0.218 | 0.83 [0.57, 1.21] | 0.330 |
| **Profession** |  |  |  |  |
| Non-health care sector | 1.00 |  | 1.00 |  |
| Health care sector | 0.94 [0.72, 1.23] | 0.664 | 1.06 [0.80, 1.40] | 0.688 |
| **Number living together** |  |  |  |  |
| < 3 people | 1.00 |  | 1.00 |  |
| 4-6 people | 1.27 [0.97, 1.67] | 0.080 | 1.23 [0.93, 1.64] | 0.150 |
| 6+ | 1.35 [0.96, 1.89] | 0.086 | 1.28 [0.89, 1.84] | 0.175 |
| **Knowledge of common symptoms** |  |  |  |  |
| **Fever** |  |  |  |  |
| No | 1.00 |  | 1.00 |  |
| Yes | 1.89 [0.84, 4.24] | 0.120 | 1.31 [0.60, 2.88] | 0.490 |
| **Fatigue** |  |  |  |  |
| No | 1.00 |  | 1.00 |  |
| Yes | 0.94 [0.71, 1.26] | 0.690 | 0.81 [0.60, 1.10] | 0.180 |
| **Dry cough** |  |  |  |  |
| No | 1.00 |  | 1.00 |  |
| Yes | 1.44 [0.71, 2.94] | 0.310 | 0.85 [0.44, 1.65] | 0.640 |
| **Sore throat** |  |  |  |  |
| No | 1.00 |  | 1.00 |  |
| Yes | 1.45 [1.04, 2.01] | 0.030 | 1.52 [1.06, 2.18] | 0.030 |
| **Unlike cold symptoms** |  |  |  |  |
| No | 1.00 |  | 1.00 |  |
| Yes | 0.79 [0.63, 0.98] | 0.03 | 0.78 [0.62, 0.98] | 0.03 |
| **Compliance to mitigation practices** |  |  |  |  |
| **Practiced Self Isolation** |  |  |  |  |
| No | 1.00 |  | 1.00 |  |
| Yes | 0.86 [0.68, 1.09] | 0.219 | 0.91 [0.71, 1.17] | 0.481 |
| **Home quarantined due to COVID-19** |  |  |  |  |
| No | 1.00 |  | 1.00 |  |
| Yes | 1.07 [0.86, 1.34] | 0.545 | 1.06 [0.84, 1.35] | 0.629 |
| **Gone to crowded place including religious events** |  |  |  |  |
| No | 1.00 |  | 1.00 |  |
| Yes | 1.27 [1.02, 1.59] | 0.034 | 1.34 [1.06, 1.69] | 0.015 |
| **Wore Facemask outside** |  |  |  |  |
| No | 1.00 |  | 1.00 |  |
| Yes | 1.15 [0.90, 1.46] | 0.270 | 1.35 [1.03, 1.76] | 0.027 |
| **Hand washing/used hand sanitizer** |  |  |  |  |
| No | 1.00 |  | 1.00 |  |
| Yes | 0.95 [0.75, 1.19] | 0.649 | 1.10 [0.86, 1.42] | 0.449 |
| **Perceived risk** |  |  |  |  |
| **Becoming infected** |  |  |  |  |
| High | 1.00 |  | 1.00 |  |
| Not high | 1.11 [0.89, 1.39] | 0.378 | 0.90 [0.71, 1.14] | 0.372 |
| **Becoming severely infected** |  |  |  |  |
| High | 1.00 |  | 1.00 |  |
| Not high | 0.98 [0.76, 1.26] | 0.884 | 0.76 [0.59, 0.99] | 0.040 |
| **Dying from the infection** |  |  |  |  |
| High | 1.00 |  | 1.00 |  |
| Not high | 0.92 [0.69, 1.21] | 0.534 | 0.86 [0.64, 1.15] | 0.318 |
| **How worried are you because of COVID-19?** |  |  |  |  |
| Worried | 1.00 |  | 1.00 |  |
| Not worried | 0.81 [0.65, 1.01] | 0.059 | 0.65 [0.51, 0.82] | <0.001 |
| **If COVID-19 continues, you or family would be directly affected?** |  |  |  |  |
| Concerned | 1.00 |  | 1.00 |  |
| Not concerned | 1.08 [0.69, 1.70] | 0.730 | 0.69 [0.40, 1.18] | 0.177 |
| **COVID-19 will continue in your country?** |  |  |  |  |
| Likely | 1.00 |  | 1.00 |  |
| not likely | 1.80 [1.43, 2.26] | <0.001 | 1.91 [1.50, 2.44] | <0.001 |

^OR, Odds Ratio; CI, Confidence Interval^
