# Supplementary material for: Misinformation About COVID-19 in Sub-Saharan Africa: Evidence from a Cross-Sectional Survey
Source: Health Secur. 2021 Feb 18;19(1):44–56. doi: 10.1089/hs.2020.0202 (PMC9347271; doi:10.1089/hs.2020.0202)
Supplement: Supplemental data [file Supp_Table2.docx]

Supplementary Table 2. Univariate analysis of factors associated with the misconception that COVID-19 infection has less effects on Blacks than on Whites

| **Variables** | **Neutral** | | **Agree** | |
| --- | --- | --- | --- | --- |
|  | **Unadjusted OR [95% CI]** | **P-value** | **Unadjusted OR [95% CI]** | **P-value** |
| **Age category (years)** |  |  |  |  |
| 18-28 | 1.00 |  | 1.00 |  |
| 29-38 | 1.06 [0.82, 1.38] | 0.640 | 0.71 [0.51, 1.00] | 0.051 |
| 39-48 | 0.76 [0.57, 1.02] | 0.064 | 0.57 [0.40, 0.83] | 0.003 |
| 49+ | 0.65 [0.45, 0.93] | 0.017 | 0.51 [0.32, 0.81] | 0.005 |
| **Sex** |  |  |  |  |
| Males | 1.00 |  | 1.00 |  |
| Females | 0.95 [0.77, 1.18] | 0.616 | 0.88 [0.67, 1.16] | 0.371 |
| **Sub-region** |  |  |  |  |
| Southern Africa | 1.00 |  | 1.00 |  |
| Central Africa | 1.32 [0.93, 1.88] | 0.12 | 1.46 [0.93, 2.28] | 0.096 |
| East Africa | 1.36 [0.97, 1.90] | 0.075 | 2.25 [1.54, 3.28] | <0.001 |
| West Africa | 1.19 [0.91, 1.56] | 0.195 | 0.87 [0.59, 1.28] | 0.487 |
| **Region of residence** |  |  |  |  |
| Africa | 1.00 |  | 1.00 |  |
| Diaspora | 1.28 [0.85, 1.91] | 0.235 | 1.51 [0.93, 2.45] | 0.099 |
| **Employment status** |  |  |  |  |
| Employed | 1.00 |  | 1.00 |  |
| Unemployed | 1.21 [0.97, 1.52] | 0.089 | 1.56 [1.18, 2.07] | 0.002 |
| **Marital Status** |  |  |  |  |
| Married | 1.00 |  | 1.00 |  |
| Not married | 1.24 [1.00, 1.53] | 0.051 | 1.36 [1.03, 1.79] | 0.031 |
| **Religion** |  |  |  |  |
| Christianity | 1.00 |  | 1.00 |  |
| Others | 0.61 [0.43, 0.86] | 0.005 | 0.47 [0.28, 0.78] | 0.004 |
| **Highest level of Education** |  |  |  |  |
| Postgraduate Degree (Masters /PhD) | 1.00 |  | 1.00 |  |
| Bachelor’s degree | 1.5 [1.19, 1.91] | 0.001 | 1.46 [1.08, 1.99] | 0.015 |
| Secondary/Primary | 1.08 [0.75, 1.55] | 0.687 | 1.19 [0.76, 1.89] | 0.449 |
| **Profession** |  |  |  |  |
| Non-health care sector | 1.00 |  | 1.00 |  |
| Health care sector | 0.84 [0.65, 1.09] | 0.194 | 0.92 [0.66, 1.29] | 0.632 |
| **Number living together** |  |  |  |  |
| < 3 people | 1.00 |  | 1.00 |  |
| 4-6 people | 1.29 [0.99, 1.69] | 0.063 | 1.17 [0.83, 1.66] | 0.357 |
| 6+ | 1.31 [0.94, 1.83] | 0.114 | 1.41 [0.93, 2.13] | 0.102 |
| **Knowledge of symptoms** |  |  |  |  |
| **Fever** |  |  |  |  |
| No | 1.00 |  | 1.00 |  |
| Yes | 0.44 (0.21, 0.91) | 0.030 | 0.46 (0.18, 1.14) | 0.090 |
| **Fatigue** |  |  |  |  |
| No | 1.00 |  | 1.00 |  |
| Yes | 0.83 (0.63, 1.10) | 0.191 | 0.76 (0.54, 1.08) | 0.124 |
| **Dry cough** |  |  |  |  |
| No | 1.00 |  | 1.00 |  |
| Yes | 0.74 (0.40, 1.37) | 0.344 | 1.08 (0.44, 2.64) | 0.873 |
| **Sore throat** |  |  |  |  |
| No | 1.00 |  | 1.00 |  |
| Yes | 0.89 (0.65, 1.24) | 0.510 | 1.09 (0.70, 1.69) | 0.702 |
| **Unlike cold symptoms** |  |  |  |  |
| No | 1.00 |  | 1.00 |  |
| Yes | 0.97 (0.79, 1.20) | 0.791 | 0.84 (0.64, 1.10) | 0.201 |
| **Compliance to mitigation practices** |  |  |  |  |
| **Practiced Self Isolation** |  |  |  |  |
| No | 1.00 |  | 1.00 |  |
| Yes | 0.94 [0.75, 1.19] | 0.604 | 0.9 [0.66, 1.21] | 0.473 |
| **Home quarantined due to COVID-19** |  |  |  |  |
| No | 1.00 |  | 1.00 |  |
| Yes | 0.93 [0.75, 1.16] | 0.530 | 0.88 [0.66, 1.17] | 0.368 |
| **Gone to crowded place including religious events** |  |  |  |  |
| No | 1.00 |  | 1.00 |  |
| Yes | 1.5 [1.22, 1.86] | <0.001 | 1.69 [1.29, 2.22] | <0.001 |
| **Wore Facemask outside** |  |  |  |  |
| No | 1.00 |  | 1.00 |  |
| Yes | 0.91 [0.72, 1.16] | 0.444 | 0.96 [0.70, 1.30] | 0.772 |
| **Hand washing/used hand sanitizer** |  |  |  |  |
| No | 1.00 |  | 1.00 |  |
| Yes | 0.73 [0.59, 0.92] | 0.006 | 0.6 [0.45, 0.79] | <0.001 |
| **Perceived risk** |  |  |  |  |
| **Becoming infected** |  |  |  |  |
| High | 1.00 |  | 1.00 |  |
| Not high | 0.99 [0.79, 1.23] | 0.928 | 1.12 [0.84, 1.50] | 0.433 |
| **Becoming severely infected** |  |  |  |  |
| High | 1.00 |  | 1.00 |  |
| Not high | 1.00 [0.79, 1.28] | 0.979 | 1.09 [0.79, 1.50] | 0.587 |
| **Dying from the infection** |  |  |  |  |
| High | 1.00 |  | 1.00 |  |
| Not high | 0.99 [0.76, 1.30] | 0.973 | 1.01 [0.71, 1.44] | 0.941 |
| **How worried are you because of COVID-19?** |  |  |  |  |
| Worried | 1.00 |  | 1.00 |  |
| Not worried | 1.05 [0.85, 1.30] | 0.653 | 1.42 [1.08, 1.87] | 0.013 |
| **If COVID-19 continues, you or family would be directly affected?** |  |  |  |  |
| Concerned | 1.00 |  | 1.00 |  |
| Not concerned | 1.35 [0.86, 2.12] | 0.187 | 1.30 [0.73, 2.31] | 0.377 |
| **COVID-19 will continue in your country?** |  |  |  |  |
| Likely | 1.00 |  | 1.00 |  |
| not likely | 1.88 [1.50, 2.34] | <0.001 | 2.58 [1.95, 3.41] | <0.001 |

^OR, Odds Ratio; CI, Confidence Interval^
