# Supplementary material for: Misinformation About COVID-19 in Sub-Saharan Africa: Evidence from a Cross-Sectional Survey
Source: Health Secur. 2021 Feb 18;19(1):44–56. doi: 10.1089/hs.2020.0202 (PMC9347271; doi:10.1089/hs.2020.0202)
Supplement: Supplemental data [file Supp_Table3.docx]

Supplementary Table 3. Univariate analysis of factors associated with the misconception that COVID-19 is designed to reduce the world population

| **Variables** | **Neutral** | | **Agree** | |
| --- | --- | --- | --- | --- |
|  | **Unadjusted OR [95% CI]** | **P-value** | **Unadjusted OR [95% CI]** | **P-value** |
| **Age category (years)** |  |  |  |  |
| 18-28 | 1.00 |  | 1.00 |  |
| 29-38 | 0.86 [0.66, 1.11] | 0.251 | 0.38 [0.27, 0.52] | <0.001 |
| 39-48 | 0.59 [0.45, 0.77] | <0.001 | 0.26 [0.18, 0.37] | <0.001 |
| 49+ | 0.55 [0.39, 0.76] | <0.001 | 0.20 [0.12, 0.32] | <0.001 |
| **Sex** |  |  |  |  |
| Males | 1.00 |  | 1.00 |  |
| Females | 1.18 [0.96, 1.45] | 0.121 | 1.79 [1.39, 2.31] | <0.001 |
| **Sub-region** |  |  |  |  |
| Southern Africa | 1.00 |  | 1.00 |  |
| Central Africa | 1.14 [0.81, 1.62] | 0.447 | 1.26 [0.83, 1.93] | 0.281 |
| East Africa | 1.60 [1.14, 2.22] | 0.006 | 2.12 [1.45, 3.10] | <0.001 |
| West Africa | 0.97 [0.75, 1.26] | 0.814 | 0.94 [0.68, 1.31] | 0.725 |
| **Region of residence** |  |  |  |  |
| Africa | 1.00 |  | 1.00 |  |
| Diaspora | 0.91 [0.62, 1.34] | 0.636 | 0.84 [0.51, 1.37] | 0.484 |
| **Employment status** |  |  |  |  |
| Employed | 1.00 |  | 1.00 |  |
| Unemployed | 1.67 [1.33, 2.09] | <0.001 | 3.30 [2.54, 4.30] | <0.001 |
| **Marital Status** |  |  |  |  |
| Married | 1.00 |  | 1.00 |  |
| Not married | 1.35 [1.10, 1.65] | 0.004 | 2.70 [2.07, 3.53] | <0.001 |
| **Religion** |  |  |  |  |
| Christianity | 1.00 |  | 1.00 |  |
| Others | 0.66 [0.47, 0.92] | 0.013 | 1.04 [0.72, 1.50] | 0.843 |
| **Highest level of Education** |  |  |  |  |
| Postgraduate Degree (Masters /PhD) | 1.00 |  | 1.00 |  |
| Bachelor’s degree | 1.63 [1.31, 2.04] | <0.001 | 2.75 [2.03, 3.74] | <0.001 |
| Secondary/Primary | 1.37 [0.97, 1.95] | 0.076 | 3.63 [2.41, 5.47] | <0.001 |
| **Profession** |  |  |  |  |
| Non-health care sector | 1.00 |  | 1.00 |  |
| Health care sector | 1.07 [0.83, 1.37] | 0.621 | 0.92 [0.67, 1.25] | 0.577 |
| **Number living together** |  |  |  |  |
| < 3 people | 1.00 |  | 1.00 |  |
| 4-6 people | 1.26 [0.98, 1.63] | 0.075 | 0.90 [0.66, 1.22] | 0.501 |
| 6+ | 1.33 [0.96, 1.83] | 0.082 | 1.04 [0.71, 1.53] | 0.833 |
| **Knowledge of symptoms** |  |  |  |  |
| **Fever** |  |  |  |  |
| No | 1.00 |  | 1.00 |  |
| Yes | 0.65 (0.31, 1.39) | 0.272 | 0.54 (0.22, 1.23) | 0.143 |
| **Fatigue** |  |  |  |  |
| No | 1.00 |  | 1.00 |  |
| Yes | 1.15 (0.87, 1.51) | 0.321 | 1.78 (0.58, 1.09) | 0.160 |
| **Dry cough** |  |  |  |  |
| No | 1.00 |  | 1.00 |  |
| Yes | 0.79 (0.42, 1.51) | 0.481 | 0.66 (0.31, 1.39) | 0.271 |
| **Sore throat** |  |  |  |  |
| No | 1.00 |  | 1.00 |  |
| Yes | 1.02 (0.75, 1.39) | 0.902 | 1.20 (0.80, 1.79) | 0.381 |
| **Unlike cold symptoms** |  |  |  |  |
| No | 1.00 |  | 1.00 |  |
| Yes | 0.86 (0.70, 1.05) | 0.154 | 1.18 (0.92, 1.52) | 0.192 |
| **Compliance to mitigation practices** |  |  |  |  |
| **Practiced Self Isolation** |  |  |  |  |
| No | 1.00 |  | 1.00 |  |
| Yes | 1.03 [0.82, 1.29] | 0.824 | 1.69 [1.30, 2.21] | <0.001 |
| **Home quarantined due to COVID-19** |  |  |  |  |
| No | 1.00 |  | 1.00 |  |
| Yes | 1.09 [0.88, 1.35] | 0.413 | 1.60 [1.24, 2.07] | <0.001 |
| **Gone to crowded place including religious events** |  |  |  |  |
| No | 1.00 |  | 1.00 |  |
| Yes | 1.47 [1.20, 1.80] | <0.001 | 1.30 [1.01, 1.67] | 0.044 |
| **Wore Facemask outside** |  |  |  |  |
| No | 1.00 |  | 1.00 |  |
| Yes | 0.92 [0.73, 1.16] | 0.488 | 1.18 [0.88, 1.58 | 0.277 |
| **Hand washing/used hand sanitizer** |  |  |  |  |
| No | 1.00 |  | 1.00 |  |
| Yes | 0.74 [0.60, 0.92] | 0.007 | 0.67 [0.52, 0.87] | 0.003 |
| **Perceived risk** |  |  |  |  |
| **Becoming infected** |  |  |  |  |
| High | 1.00 |  | 1.00 |  |
| Not high | 1.06 [0.86, 1.31] | 0.608 | 1.24 [0.95, 1.61] | 0.118 |
| **Becoming severely infected** |  |  |  |  |
| High | 1.00 |  | 1.00 |  |
| Not high | 1.05 [0.83, 1.33] | 0.659 | 1.11 [0.83, 1.49] | 0.466 |
| **Dying from the infection** |  |  |  |  |
| High | 1.00 |  | 1.00 |  |
| Not high | 1.09 [0.84, 1.41] | 0.523 | 1.12 [0.81, 1.55] | 0.482 |
| **How worried are you because of COVID-19?** |  |  |  |  |
| Worried | 1.00 |  | 1.00 |  |
| Not worried | 0.98 [0.80, 1.21] | 0.860 | 1.03 [0.80, 1.33] | 0.836 |
| **If COVID-19 continues, you or family would be directly affected?** |  |  |  |  |
| Concerned | 1.00 |  | 1.00 |  |
| Not concerned | 1.23 [0.78, 1.92] | 0.368 | 1.22 [0.71, 2.11] | 0.477 |
| **COVID-19 will continue in your country?** |  |  |  |  |
| Likely | 1.00 |  | 1.00 |  |
| not likely | 1.96 [1.58, 2.44] | <0.001 | 1.55 [1.19, 2.03] | 0.001 |

^OR, Odds Ratio; CI, Confidence Interval^
