# Supplementary material for: Misinformation About COVID-19 in Sub-Saharan Africa: Evidence from a Cross-Sectional Survey
Source: Health Secur. 2021 Feb 18;19(1):44–56. doi: 10.1089/hs.2020.0202 (PMC9347271; doi:10.1089/hs.2020.0202)
Supplement: Supplemental data [file Supp_Table4.docx]

Supplementary Table 4. Univariate analysis of factors associated with the misconception that the ability to hold one’s breath for 10 seconds means you do not have COVID-19

| Variables | **Neutral** | | **Agree** | |
| --- | --- | --- | --- | --- |
|  | **Unadjusted OR [95% CI]** | **P-value** | **Unadjusted OR [95% CI]** | **P-value** |
| **Age category (years)** |  |  |  |  |
| 18-28 | 1.00 |  | 1.00 |  |
| 29-38 | 1.01 [0.77, 1.31] | 0.961 | 0.91 [0.67, 1.24] | 0.561 |
| 39-48 | 1.23 [0.93, 1.63] | 0.150 | 1.16 [0.84, 1.60] | 0.355 |
| 49+ | 0.71 [0.50, 1.02] | 0.063 | 0.92 [0.63, 1.35] | 0.686 |
| **Sex** |  |  |  |  |
| Males | 1.00 |  | 1.00 |  |
| Females | 0.80 [0.65, 0.99] | 0.047 | 0.97 [0.76, 1.24] | 0.824 |
| **Sub-region** |  |  |  |  |
| Southern Africa | 1.00 |  | 1.00 |  |
| Central Africa | 1.06 [0.74, 1.50] | 0.761 | 0.62 [0.39, 0.97] | 0.035 |
| East Africa | 1.31 [0.94, 1.83] | 0.113 | 1.14 [0.78, 1.66] | 0.492 |
| West Africa | 0.93 [0.71, 1.22] | 0.606 | 0.76 [0.56, 1.04] | 0.091 |
| **Region of residence** |  |  |  |  |
| Africa | 1.00 |  | 1.00 |  |
| Diaspora | 0.94 [0.64, 1.40] | 0.776 | 0.70 [0.43, 1.15] | 0.161 |
| **Employment status** |  |  |  |  |
| Employed | 1.00 |  | 1.00 |  |
| Unemployed | 1.10 [0.88, 1.38] | 0.386 | 1.04 [0.80, 1.34] | 0.786 |
| **Marital Status** |  |  |  |  |
| Married | 1.00 |  | 1.00 |  |
| Not married | 0.99 [0.80, 1.23] | 0.952 | 0.93 [0.73, 1.18] | 0.544 |
| **Religion** |  |  |  |  |
| Christianity | 1.00 |  | 1.00 |  |
| Others | 0.85 [0.61, 1.19] | 0.340 | 0.91 [0.63, 1.32] | 0.633 |
| **Highest level of Education** |  |  |  |  |
| Postgraduate Degree (Masters /PhD) | 1.00 |  | 1.00 |  |
| Bachelor’s degree | 1.19 [0.94, 1.50] | 0.142 | 1.12 [0.86, 1.46] | 0.405 |
| Secondary/Primary | 0.85 [0.59, 1.21] | 0.361 | 0.96 [0.64, 1.42] | 0.824 |
| **Profession** |  |  |  |  |
| Non-health care sector | 1.00 |  | 1.00 |  |
| Health care sector | 1.09 [0.84, 1.41] | 0.511 | 1.09 [0.81, 1.46] | 0.575 |
| **Number living together** |  |  |  |  |
| < 3 people | 1.00 |  | 1.00 |  |
| 4-6 people | 1.30 [0.99, 1.69] | 0.054 | 1.26 [0.94, 1.69] | 0.122 |
| 6+ | 1.24 [0.89, 1.72] | 0.197 | 0.91 [0.62, 1.33] | 0.617 |
| **Knowledge of symptoms** |  |  |  |  |
| **Fever** |  |  |  |  |
| No | 1.00 |  | 1.00 |  |
| Yes | 1.25[0.59, 2.66] | 0.55 | 1.33[0.55, 3.19] | 0.52 |
| **Fatigue** |  |  |  |  |
| No | 1.00 |  | 1.00 |  |
| Yes | 1.13 [0.86, 1.50] | 0.382 | 0.94 [0.69, 1.28] | 0.686 |
| **Dry cough** |  |  |  |  |
| No | 1.00 |  | 1.00 |  |
| Yes | 0.95 [0.49, 1.83] | 0.872 | 0.84 [0.41, 1.74] | 0.654 |
| **Sore throat** |  |  |  |  |
| No | 1.00 |  | 1.00 |  |
| Yes | 1.04 [0.75, 1.44] | 0.810 | 1.22 (0.83, 1.78) | 0.324 |
| **Unlike cold symptoms** |  |  |  |  |
| No | 1.00 |  | 1.00 |  |
| Yes | 0.72 [0.58, 0.89] | 0.002 | 0.76 [0.60, 0.97] | 0.031 |
| **Compliance to mitigation practices** |  |  |  |  |
| **Practiced Self Isolation** |  |  |  |  |
| No | 1.00 |  | 1.00 |  |
| Yes | 0.99 [0.79, 1.24] | 0.930 | 0.85 [0.65., 1.10] | 0.218 |
| **Home quarantined due to COVID-19** |  |  |  |  |
| No | 1.00 |  | 1.00 |  |
| Yes | 1.12 [0.90, 1.39] | 0.311 | 1.02 [0.80, 1.31] | 0.854 |
| **Gone to crowded place including religious events** |  |  |  |  |
| No | 1.00 |  | 1.00 |  |
| Yes | 1.34 [1.08, 1.66] | 0.007 | 1.15 [0.90, 1.46] | 0.268 |
| **Wore Facemask outside** |  |  |  |  |
| No | 1.00 |  | 1.00 |  |
| Yes | 0.92 [0.72, 1.16] | 0.470 | 1.1 [0.84, 1.45] | 0.490 |
| **Hand washing/used hand sanitizer** |  |  |  |  |
| No | 1.00 |  | 1.00 |  |
| Yes | 0.87 [0.69, 1.09] | 0.223 | 1.19 [0.92, 1.55] | 0.192 |
| **Perceived risk** |  |  |  |  |
| **Becoming infected** |  |  |  |  |
| High | 1.00 |  | 1.00 |  |
| Not high | 1.30 [1.04, 1.62] | 0.021 | 1.13 [0.88, 1.44] | 0.339 |
| **Becoming severely infected** |  |  |  |  |
| High | 1.00 |  | 1.00 |  |
| Not high | 1.16 [0.91, 1.49] | 0.227 | 0.91 [0.69, 1.18] | 0.464 |
| **Dying from the infection** |  |  |  |  |
| High | 1.00 |  | 1.00 |  |
| Not high | 1.11 [0.84, 1.46] | 0.463 | 0.85 [0.64, 1.15] | 0.293 |
| **How worried are you because of COVID-19?** |  |  |  |  |
| Worried | 1.00 |  | 1.00 |  |
| Not worried | 0.89 [0.72, 1.10] | 0.286 | 0.8 [0.63, 1.03] | 0.079 |
| **If COVID-19 continues, you or family would be directly affected?** |  |  |  |  |
| Concerned | 1.00 |  | 1.00 |  |
| Not concerned | 1.45 [0.94, 2.24] | 0.093 | 0.71 [0.39, 1.29] | 0.263 |
| **COVID-19 will continue in your country?** |  |  |  |  |
| Likely | 1.00 |  | 1.00 |  |
| not likely | 2.20 [1.76, 2.75] | <0.001 | 1.84 [1.43, 2.37] | <0.001 |

^OR, Odds Ratio; CI, Confidence Interval^
